# Health-seeking behaviour of breast cancer patients receiving care at a tertiary institution in Ghana

**DOI:** 10.3332/ecancer.2024.1756

**Published:** 2024-09-06

**Authors:** Florence Dedey, Josephine Nsaful, Kirstyn E Brownson, Ruth Y Laryea, Nathaniel Coleman, John Tetteh, Joe-Nat Clegg-Lamptey, Benedict N L Calys-Tagoe

**Affiliations:** 1 Department of Surgery, University of Ghana Medical School, Korle Bu, PO Box GP 4236, Accra, Ghana; 2 Department of Surgery, Korle Bu Teaching Hospital, PO Box 77, Accra, Ghana; 3 Department of Surgery, University of Utah School of Medicine, Salt Lake City, UT 84132, USA; 4 Huntsman Cancer Institute, Salt Lake City, UT 84112, USA; 5 Department of Medicine and Therapeutics, University of Ghana Medical School, Korle Bu, PO Box GP 4236, Accra, Ghana; 6 Department of Obstetrics and Gynaecology, University of Ghana Medical School, Korle Bu, PO Box GP 4236, Accra, Ghana; 7 Department of Community Health, University of Ghana Medical School, Korle Bu, PO Box GP 4236, Accra, Ghana

**Keywords:** healthcare delay, health seeking behaviour, cancer care, breast cancer, knowledge

## Abstract

**Background:**

Breast cancer incidence rates are rising in Africa and mortality is highest in West Africa. Reasons for poor survival are multifactorial but delays in seeking appropriate health care result in late presentation which contributes significantly to poor outcomes. Total delays of more than 3 months have been associated with advanced stage at presentation and poorer survival.

**Method:**

A cross-sectional design was used to assess delays in health-seeking behaviour in consecutive breast cancer patients receiving treatment at Korle Bu Teaching Hospital (KBTH) from January to December 2022 using a structured, interviewer-administered questionnaire. Data were gathered to assess health-seeking behaviour in relation to delays in a presentation to a health care facility, and factors that may have influenced the delays. Statistical analysis was done using descriptive and inferential analyses.

**Results:**

The study involved 636 participants with a mean age and SD of 52.6 ± 12 years. Most participants were diagnosed with Stage 3 or 4 breast cancer (56.5%). Ninety percent of participants had visited at least one health facility prior to seeking care at KBTH. Forty-two percent of the participants sought care at a health facility less than a month after noticing symptoms of breast cancer while 34.4% did so greater than 3 months after noticing symptoms. Delays showed a significant association with age, marital status, educational level, average monthly income and cancer stage (*p* < 0.05). Common reasons for delays were lack of knowledge of breast cancer signs and/or symptoms (47%), advice from family and friends (15%), financial difficulties (9%), seeking alternate treatments (7%), competing priorities (6%) and indifference (5%).

**Conclusion:**

Lack of knowledge about breast cancer was a major cause of delay in seeking health care in this study. Education should specifically target knowledge about breast cancer and the need for appropriate and timely health seeking.

## Introduction

Breast cancer is responsible for the majority of cancers in women worldwide. Although the incidence of breast cancer is lower in Sub-Saharan Africa (SSA) compared to North America, rates are rising across Africa, including in Ghana [1–3]. It is expected that incidence and mortality rates of breast cancer will double for low human development index countries by 2040 [4]. Breast cancer mortality is high in most low-and-middle-income countries (LMICs) with Western Africa having the highest mortality rate of >20/100,000. The mortality-to-incidence ratio of breast cancer in Western Africa is 0.54 as compared to 0.13 in Northern America [1]. The 5-year survival of breast cancer in Western Africa is 35%–48% compared to greater than 80% in high-income countries [3–7]. In Ghana, because breast cancer is the most commonly diagnosed cancer and is responsible for most cancer-related deaths in women [1, 8], it is now considered a public health burden [4].

Reasons for poor survival rates amongst patients with breast cancer in Ghana are multifactorial including lack of national screening programs for early detection, late presentation with advanced disease and limitations in accessing effective treatment. Delays in the presentation can be influenced by patient health-seeking behaviour and contribute significantly to poor outcomes. Patient health-seeking behaviour is influenced by many factors including individual predisposing (sociodemographic, fund of knowledge about disease and attitudes towards health services), enabling (availability and accessibility of health services) and need (reasons for seeking health care eg perception of illness severity) factors [9–11]. These factors can predispose patients to delays in seeking medical care [12]. Delays in treating breast cancer can be due to patient-driven delays or healthcare system/provider delays, together making up the total delay. The patient delay is the time interval between first noting symptoms of disease and presentation at a healthcare facility while healthcare system/provider delay is usually considered to be a prolonged interval between the patient’s first visit to a healthcare worker and initiation of treatment [12–14]. Total delays of more than 3 months have been associated with advanced stage at presentation and poorer survival [15, 16]. Waiting for more than 3 months after noting symptoms of breast cancer before seeking care has therefore been considered as a significant patient delay [10, 12, 14, 17–22]. Median patient delay in LMICs has been found to be 1.4–2.9 times higher than in high-income countries [12].

There is evidence that early detection and treatment can result in a cure for breast cancer. However, most cases in Ghana present with late-stage disease where a cure is less likely [23]. Seventy-seven percent of black women with breast cancer in SSA have advanced disease (Stage III or IV) at the time of presentation to a healthcare facility [24]. Patients in Ghana are no exception and frequently present to healthcare providers late with large tumours, lymph node metastases and distant metastatic disease [23]. Additionally, breast cancer patients in SSA have a higher percentage of more aggressive cancers with poorer biological features than is seen in high-income countries, making early presentation to a medical facility even more imperative for patient survival [25].

Knowing how long it takes patients to seek care at a healthcare facility after noticing symptoms of breast cancer, and understanding the factors that influence this health-seeking behaviour in breast cancer patients, will inform interventions to reduce delays and promote good health-seeking behaviour to ultimately decrease late-stage diagnosis and improve patient survival.

## Methods

A cross-sectional design using a quantitative approach was used to assess delays in health-seeking behaviour in breast cancer patients receiving treatment at Korle Bu Teaching Hospital (KBTH), the largest referral and teaching hospital in Ghana with a dedicated surgical breast unit, an oncology center, and a radiotherapy center, offering multidisciplinary breast care to patients. Consecutive females with a histologic diagnosis of breast cancer who had been receiving breast cancer treatment at the Surgical Department and/or the National Centre for Radiotherapy, Oncology and Nuclear Medicine at KBTH for at least 3 months were approached to participate in this study. Male breast cancer patients were excluded. Data were collected over a 1-year period from January 2022 through to December 2022. Written informed consent was obtained. Participants were selected from the surgical Outpatient Department (OPD), chemotherapy suite, surgical wards and the radiotherapy OPD. Measures were put in place to avoid multiple recruitment of the same individual. A structured, interviewer-administered questionnaire was used for data collection. The questionnaire was pretested among a smaller sample [26] of breast cancer patients in a nearby facility with similar patient characteristics. This helped to improve the quality of the tool as ambiguous and culturally sensitive questions were modified and made more suitable for the study population. This included gathering data to assess factors that may have caused delays in their presentation to the hospital for treatment. The interviews were conducted in person by trained research assistants.

***The main outcome measure considered was a patient delay in seeking healthcare services. Participants were asked, ‘How long after noticing your symptoms did you report to a healthcare facility?’ This was measured in weeks and converted to months. The converted continuous data were re-classified into ordinal variables: <1, 1–2, 3–6 and >6 months. For sensitivity analysis, a binary outcome assessment was conducted by generating an additional five binary outcomes, i.e., delay at 2, 3, 4, 5 and 6 months. Descriptive and inferential analyses were adopted separately. For the descriptive analysis, frequencies and percentages were used to summarize the variables. The chi-square test was used to assess differences in proportions in ordinal delay outcomes for the inferential analysis. Additionally, a regression analysis was performed, using six models comprising ordinal regression model and binary logistic regression. As the ordinal outcome of delays in seeking healthcare was the main focus, the ordinal logistic regression was applied as model 1 to assess associated outcomes. Models 2–6 were sensitivity analyses employing the binary logistics on the binary outcomes (delays at 2, 3, 4, 5 and 6 months). To select the best model from sensitivity analysis to supplement the focus model (model 1), the area under the curve post estimation was used to determine the best model. All analyses were performed using Stata 16.1 and *p* < 0.05 was deemed statistically significant.

### Ethical considerations

The KBTH Institutional Review Board reviewed and approved the study protocol (KBTH-STC/IRB/00099/2021). Written informed consent was obtained from all participants and research was conducted in line with standard research ethical principles. No participant-identifying details were used to ensure patient confidentiality.

## Results

The study involved 636 participants most of whom (58%) were greater than 50 years of age. The mean age and SD was 52.6 ± 12.1 years. Approximately 83% of the participants had at least Middle or Junior High School certificates or higher education degrees. Fifty-six percent of participants were married. The majority of the study participants (88.1%) lived in an urban area within Ghana and most were currently employed (68.9%). About one third (37.7%) of the study participants earned less than GHC500 (56 USD) a month. The majority (90%) had visited at least one healthcare facility before being seen at KBTH. More than half of the participants were diagnosed with Stage 3 or Stage 4 breast cancer (56.5%).

**Figure 1.**
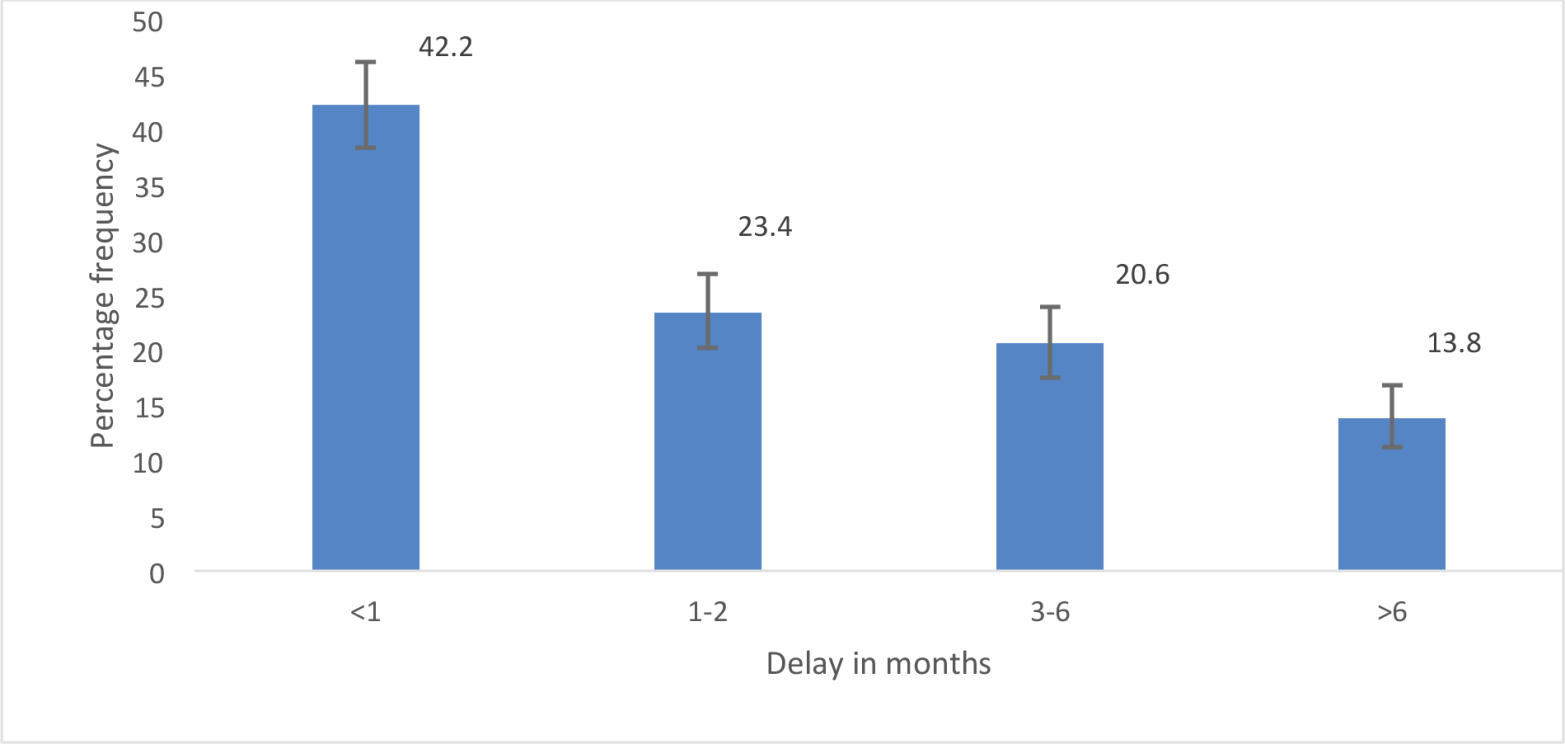
Percentage frequency of delay in health seeking among participants.

About two thirds of the participants (65.6%) sought medical care at a healthcare facility within 3 months of noticing concerning symptoms of breast cancer with 42% (*n* = 268) doing so less than a month after they noticed these symptoms. A third of the patients (34.4%), however, sought care more than 3 months after noticing breast changes (Figure 1). The median duration between noticing symptoms and seeking healthcare was 4 weeks.

The differences in the proportion of delay in health seeking after noticing symptoms were significantly associated with patient level of education, average monthly income, initial facility visited and cancer stage (*p* < 0.05) (Table 1).

Furthermore, from the focus model (Tables 2 and 3) factors including age, marital status, number of breast cancer symptoms at the time of presentation, initial facility visited, number of health facilities visited and cancer stage were significantly associated with delays in seeking healthcare for the treatment of their breast cancer. Increasing age was associated with less chance of a delay in seeking healthcare. The chance of delaying health seeking for at least 1 month was greater in divorced/separated participants compared with those who were married (aOR = 1.98; 95%CI = 1.07–3.65). Those with two or more symptoms of breast cancer at the time of presentation to a healthcare facility were 31% more likely to have presented at least 1 month later than patients presenting with 1 breast cancer symptom (OR = 1.31; 95%CI = 0.92–1.87). Study participants who initially sought care from a facility other than a healthcare facility were 2.77 times (95%CI = 1.67–4.59) more likely to have experienced at least a 1-month delay in presentation to a healthcare facility compared with those who visited a healthcare facility initially at the time of presentation. Those who did not visit any facility, healthcare or otherwise, prior to presenting at KBTH were more likely to have also experienced patient delays. Participants with Stages III and IV cancer were more likely to have experienced delays of more than 1 month compared with Stages I and II study participants (Tables 2 and 3).

Participants identified several reasons for their delay in seeking healthcare after they first noticed abnormal breast changes. Specifically, 47% reported a lack of knowledge about breast cancer symptoms as the reason for not seeking medical care sooner. Fifteen percent reported that family and/or friends advised them not to seek care. Nine percent of patients cited financial difficulties as the reason they did not seek medical care sooner while 7% named competing priorities such as work and family commitments. Finally, seven percent noted a preference for first attempting alternative treatments (including faith-based healing) prior to seeking medical care for a cure. Five percent mentioned indifference as the reason for their delay in seeking medical care (Table 4). For 12 participants who presented within 2 weeks of noticing symptoms, the reasons given for doing so within that short period were: having a good knowledge about breast cancer, wanting to know what the diagnosis was and having pain in the breast (results not shown in table).

## Discussion

Good healthcare-seeking behaviour for the treatment of breast cancer and improved breast cancer outcomes involves looking for medical care from a healthcare practitioner when faced with ill health [9] which should be done in a timely manner. An interval of more than 3 months from when the patient first notices abnormal breast symptoms to first seeking medical care has been considered a significant patient delay in many studies [10, 12, 14, 17–22]. This delay of more than 3 months has been associated with advanced breast cancer stages of diagnosis at the time of presentation [17, 18, 26] and with poorer patient outcomes [15, 16]. A similar study done in Nigeria, by Agodirin *et al* [27] reported that average tumour size increased by 0.4 cm per month over the first 12 months after noticing a lump in patients diagnosed with breast cancer. In this study, the majority (65.6%) of patients sought care from a health facility in less than 3 months. Furthermore, 42.2% of the patients sought care within 1 month of noticing symptoms which is commendable. The median duration between noticing breast symptoms and seeking appropriate health care was 4 weeks. This shows an improvement compared to the reported median duration of 34 weeks from when patients noticed symptoms to when they first presented to a healthcare facility in a study in Ghana by Clegg-Lamptey *et al* [28], performed at KBTH approximately 15 years ago. Breast cancer awareness and educational activities have been intensified in Accra and other major cities in Ghana over the last several years and could be a contributory factor to more patients seeking healthcare earlier. However, the majority of patients are still diagnosed in advanced stages with poor outcomes. This could possibly be because the patients may not be picking up the early symptoms of breast cancer. Education must therefore emphasize the earliest symptoms that can be detected by patients such as small painless lumps. Better still, consideration should be given to making screening for breast cancer available in localities with access to multidisciplinary breast cancer care such as the James Town community. Notably, a third of participants (34.4%) first presented to a health facility after 3 months from when they first noticed symptoms. This is comparable to what was reported among breast cancer patients in other LMICs where more than 35% of patients presented after 3 months from the onset of breast cancer symptoms [14, 18, 19, 21]. This lags behind what pertains in high-income countries where >60% of patients with breast cancer will have presented to a health facility, been diagnosed and started treatment in less than 3 months thereby having a total delay, rather than a patient delay, of less than 3 months [12].

The majority of patients (91%) in this study initially visited a healthcare facility upon noticing breast cancer symptoms. This was very similar to what was reported in a study from Nigeria by Agodirin *et al* [27] where 85% of breast cancer patients first sought medical care after noticing concerning symptoms, but this is not the case in all LMICs. For example, a study from Ethiopia by Hassen *et al* [19] reported that only 54% of patients first sought medical care when confronted with symptoms of breast cancer. In the latter study, rural residence was associated with a statistically significant delay in care. It is important to note that a large percentage of patients in our study were urban dwellers and lived in close proximity to a tertiary care center (KBTH) and that, thus, this data may not be able to be extended to all Ghanaians.

About 65% of the participants in our study made the decision to seek medical care of their own volition because of their belief that the healthcare system could solve their medical problems. In a study on health-seeking behaviour in communities in Sunyani, Ghana, by Abor and Ghartey [29], it was found that 92% of the participants when unwell sought care at hospitals due to their confidence in the ability of healthcare facilities to accurately diagnosis and treat patients. In our study, about 90% of the participants had visited at least one health facility prior to being seen at the KBTH. This is to be expected as KBTH is a referral center, so most patients are seen on referral from other facilities. However, over a third of the patients had visited two or three facilities prior to being referred to KBTH. If a patient is dissatisfied with healthcare recommendations at one facility and then decides to seek additional opinions at other facilities, this could delay the time to initiation of treatment. Alternatively, that patients were seen at 2–3 facilities prior to being seen at KBTH could be due to the existing referral system in Ghana where patients are usually referred from a primary care facility to a district hospital and then to a regional/tertiary/cancer center. This could result in delays in having the appropriate treatment started if the referral system is not efficient. Agodirin *et al* [27] reported that 27% of the respondents with breast cancer had visited two or more health practitioners before being referred to a specialist. In that study, although most of the respondents sought medical care within 2 months after noticing their symptoms, about 66% had more than a 3-month delay from symptom detection to the first specialist appointment. There was a further delay from the first health practitioner visit to specialist consultation which was actually the longest delay in the patient’s total time to treatment initiation [27]. A breast cancer study by Hassen *et al* [19] reported similar delays to hospital referral after the patient first reported to a healthcare practitioner in 50% of patients. Furthermore, about a third of patients had more than 3 health care visits prior to hospital referral for treatment [19]. This study found that those who had not visited any facility prior to being seen at KBTH were more likely to have had patient delays. This could be explained by the fact that those who come in straight to KBTH without a referral from another facility are likely to have come in as emergency cases with an advanced stage of breast cancer.

In this study, those who first visited a health facility after noticing breast symptoms had a statistically significant less delay compared to those who first sought non-medical care. Brinton *et al* [26] also report that not seeking first assistance from a doctor or nurse was related to larger tumour size at presentation and delays in seeking care. Patients who presented with stage III and IV breast cancer showed a statistically significant delay and this can be explained by the fact that delays in presentation result in advanced disease. Similarly, those with two or more symptoms at presentation were likely to have delayed in seeking health care, as more symptoms may develop with time as the cancer advances.

Dill [11] discussed individual sociodemographic, economic, illness and its perceived severity factors influencing health-seeking behaviour in Uganda. Similar factors were found to influence health-seeking behaviour in this study. Sociodemographic factors such as the educational level of the patients, age and marital status were found to be significantly associated with delays. Patients with a higher educational level, i.e., tertiary education were more likely to seek health care within a month of noticing symptoms as more educated patients are likely to have increased knowledge about health. Similar findings of patient delays being associated with low educational levels have also been reported in other studies [14, 17, 19, 26]. The association of age with delays has not been consistent as other studies have found both young and old age to be significantly associated with delays [14, 17, 22]. In this study, older women were less likely to delay seeking healthcare. Participants in this study who were divorced or separated had a greater chance of at least a 1-month delay in health seeking as compared to their married counterparts. Being unmarried has been found in other studies to be significantly associated with delays [19, 22]. This could be explained by the fact that unmarried patients are less likely to have the support needed to seek health care speedily. Place of residence in this study was not significantly associated with delays as also documented in some other studies [19].

Income level as an enabling factor was also found to be significantly associated with delays in this study. A monthly income of more than GHC 5,000 (USD 556) which is considered a high wage in Ghana) was associated with seeking care within 1 month of symptom detection. Having a higher income makes healthcare more affordable and hence more accessible. Low income has also been reported to be associated with delays in a study in Indonesia by Hutajalu *et al* [18]. A study in Gaza by Abo Al-Shiekh *et al* [20] reported that low income was not associated with delays and this was possibly attributed to the widespread availability of health insurance. In Ghana, Adei *et al* [9] reported that participants were less likely to adopt good health-seeking behaviour if they were not actively on health insurance.

Need factors such as the perceived severity of illness were found to influence health-seeking behaviour in breast cancer patients. The commonest reasons given for delays in seeking health care included ignorance of the symptoms of breast cancer which was cited by about half (47%) of the participants, followed by advice from family and friends (15%), financial difficulties (9%), seeking alternate treatment (7%), competing priorities (6%) and indifference (5%). Similar factors such as not attributing symptoms to cancer [10, 14, 17–20, 22, 27], fear of the disease and its treatment [17, 18, 22], finances [14, 27] use of alternate medicine including spiritual cures [10, 14, 19, 22] and competing interests [22] have been found to contribute to delays in seeking health care. Other reasons cited for delays included the COVID-19 restrictions during the period of the study and fear of contracting COVID-19. This study was conducted in 2022 when the COVID-19 pandemic was still on-going although it was not as severe and restrictions had largely been eased. On the other hand, this study found that for those who sought health care within 2 weeks of noticing their symptoms, reasons for doing so included being well informed about breast cancer, a desire to know what the diagnosis was and having pain in the breast. This buttresses the point that awareness and education about breast cancer needs to be intensified.

Various factors as we found, have also been found to affect health-seeking behaviours of individuals including demographic, socioeconomic and cultural factors [9, 30]. Understanding what influences delays in seeking healthcare in breast cancer patients is essential to combatting delays with the hope of making an earlier diagnosis to enable treatment at early stages where the prognosis is much more favourable. A review of health-seeking behaviour buttresses this point [30].

## Conclusion

The median duration between noticing symptoms and seeking healthcare was 4 weeks. This shows an improvement compared to the reported median duration of 34 weeks in a 2009 study in the same hospital. This improvement notwithstanding, a third of the breast cancer patients first reported to a health facility after 3 months of noticing breast symptoms. Factors associated with this delay in health seeking were increasing age, being divorced/separated, low educational level, low average monthly income, initial presentation to a non-medical facility having two or more symptoms and advanced cancer stage. The most commonly cited reasons for these delays were lack of knowledge about breast cancer symptoms, advice from family/friends not to seek medical care and inadequate finances for treatment. Having knowledge about breast cancer on the other hand was a reason cited for seeking health care early. Understanding the health-seeking behaviour and causes of the delayed presentation will inform our breast cancer educational programs and health systems management in an effort to reduce the delays that contribute to the advanced stage of presentation and resultant poor survival. Education should specifically target knowledge about breast cancer, specifically its’ early symptoms and the need for good health-seeking behaviour. Consideration should also be given to screening programs for communities with access to comprehensive breast care.

## Conflicts of interest

The authors have no conflict of interest to declare.

Kirstyn E Brownson is a representative on the ImpediMed Lymphedema Surgeon Advisory Board.

## Author contributions

BNLCT, KEB, JN, FD and JNCL conceptualised and designed the study. NC and RYL were responsible for data collection. FD, BNLCT and JT analysed and drafted the manuscript and all authors made significant intellectual contributions. All authors read and approved the final version of the manuscript.

## Figures and Tables

**Table 1.** Delays among breast cancer patients by socio-demographic characteristics.

Variable		Delay in months			Total	*p*-value
	<1	1–2	3–6	> 6		
	*n* (%)	*n* (%)	*n* (%)	*n* (%)	*N*	
Age						0.320
≤39	39 (40.2)	26 (26.8)	19 (19.6)	13 (13.4)	97	
40–49	85 (49.7)	35 (20.5)	32 (18.7)	19 (11.1)	171	
50–59	68 (36.4)	45 (24.1)	48 (25.7)	26 (13.9)	187	
60+	76 (42.0)	43 (23.8)	32 (17.7)	30 (16.6)	181	
Religion						0.869
No religion	3 (60.0)	0 (0.0)	1 (20.0)	1 (20.0)	5	
Christianity	242 (41.8)	138 (23.8)	119 (20.6)	80 (13.8)	579	
Islam	21 (42.0)	11 (22.0)	11 (22.0)	7 (14.0)	50	
Traditional/Spiritualist	2 (100.0)	0 (0.0)	0(0.0)	0(0.0)	2	
Educational level						**0.005**
No formal education	20 (37.7)	11 (20.8)	14 (26.4)	8 (15.1)	53	
Primary	21 (38.2)	12 (21.8)	16 (29.1)	6 (10.9)	55	
Middle/Junior High School	55 (34.0)	48 (29.6)	40 (24.7)	19 (11.7)	162	
Secondary/Senior High School	65 (39.9)	41 (25.1)	24 (14.7)	33 (20.3)	163	
Tertiary	107 (52.7)	37 (18.2)	37 (18.2)	22 (10.8)	203	
Marital status						0.607
Never married	39 (50.7)	16 (20.8)	13 (16.9)	9 (11.7)	77	
Married	154 (43.0)	81 (22.6)	73 (20.4)	50 (14.0)	358	
Co-habiting	3 (42.9)	3 (42.9)	1 (14.3)	0 (0.0)	7	
Divorced/Separated	31 (31.6)	27 (27.6)	26 (26.5)	14 (14.3)	98	
Widowed	41 (42.7)	22 (22.9)	18 (18.7)	15 (15.6)	96	
Place of residence						0.307
Urban	239 (42.7)	126 (22.5)	115 (20.5)	80 (14.3)	560	
Peri-urban	20 (33.3)	21 (35.0)	13 (21.7)	6 (10.0)	60	
Rural	9 (56.3)	2 (12.5)	3 (18.7)	2 (12.5)	16	
Employment status						0.780
Not employed	78 (39.4)	49 (24.7)	41 (20.7)	30 (15.2)	198	
Employed	190 (43.4)	100 (22.8)	90 (20.6)	58 (12.2)	438	
Average monthly income (GHS)						**0.010**
< 500	91 (38.2)	61 (25.6)	55 (23.1)	31 (13.0)	238	
500–1,000	59 (33.5)	52 (29.5)	34 (19.3)	31 (17.6)	176	
1,001–2,000	42 (49.4)	17 (20.0)	14 (16.5)	12 (14.1)	85	
2,001–5,000	49 (53.3)	13 (14.1)	20 (21.7)	10 (10.9)	92	
> 5,000	25 (62.5)	5 (12.5)	7 (17.5)	3 (7.5)	40	
Symptoms						0.078
1 only	216 (44.8)	111 (23.0)	91 (18.9)	64 (13.3)	482	
2+	52 (33.8)	38 (24.7)	40 (26.0)	24 (15.6)	154	
Initial facility visited						**<0.001**
Health facility	262 (45.5)	135 (23.4)	104 (18.1)	75 (15.0)	576	
Other places	6 (10.0)	14 (23.3)	27 (45.0)	13 (21.7)	60	
Number of health facilities visited prior to presentation at KBTH						0.249
None	22 (36.1)	10 (16.4)	14 (22.9)	15 (24.6)	61	
1 facility	140 (42.3)	82 (24.8)	65 (19.6)	44 (13.3)	331	
2 facilities	85 (45.2)	43 (22.9)	36 (19.2)	24 (12.8)	188	
3+ facilities	21 (37.5)	14 (25.0)	16 (28.6)	5 (8.9)	56	
Tumour stage						**<0.001**
Stage I & II	135 (54.9)	44 (31.0)	27 (22.1)	23 (29.1)	229	
Stage III & IV	111 (45.1)	98 (69.0)	95 (77.9)	56 (70.9)	360	

Significance was set at *p*-value < 0.05. All the bold values are less than 0.05 and therefore statistically significant.

**Table 2.** Multivariate ordinal and logistic regression showing factors associated with breast cancer among study participants with less than two months delay before visiting treatment facility with the onset of symptoms.

Variable	Delay in months		
	Ordinal outcome	Model performance for binary outcomes	
	Model 1: focused 1 month delay	Model 2 2 months delay	Model 3 3 months delay
		aOR (95%CI) *p*-value	aOR (95%CI) *p*-value
Age			
≤39	**Ref**	**Ref**	**Ref**
40–49	0.54 (0.32–0.91) **0.020**	0.50 (0.28–0.92) **0.024**	0.68 (0.38–0.126) 0.219
50–59	0.80 (0.48–1.34) 0.402	0.69 (0.38–1.26) 0.225	1.05 (0.57–1.94) 0.875
60+	0.59 (0.33–1.06) 0.079	0.45 (0.23–0.88) **0.019**	0.67 (0.34–1.34) 0.261
Religion			
Christianity	**Ref**	**Ref**	**Ref**
Islam	0.85 (0.46–1.54) 0.585	0.96 (0.49–1.88) 0.898	0.86 (0.42–1.76) 0.679
Educational level			
No formal education	**Ref**	**Ref**	**Ref**
Primary	1.02 (0.49–2.12) 0.966	1.47 (0.62–3.49) 0.386	1.27 (0.52–3.08) 0.598
Middle/Junior High School	1.13 (0.61–2.12) 0.698	1.46 (0.69–3.06) 0.322	1.02 (0.48–2.18) 0.960
Secondary/Senior High School	1.22 (0.64–2.32) 0.538	1.10 (0.69–2.34) 0.796	1.03 (0.47–2.24) 0.937
Tertiary	0.95 (0.48–1.89) 0.889	1.03 (0.46–2.30) 0.943	0.91 (0.39–2.09) 0.818
Marital status			
Never married	**Ref**	**Ref**	**Ref**
Married	1.53 (0.91–2.57) 0.107	1.59 (0.87–2.88) 0.130	1.41 (0.75–2.63) 0.285
Divorced/Separated	1.98 (1.07–3.65) **0.029**	1.77 (0.87–3.61) 0.116	1.79 (0.86–3.73) 0.121
Widowed	1.69 (0.88–3.26) 0.116	2.02 (0.94–4.33) 0.070	1.53 (0.69–3.36) 0.290
Place of residence			
Urban	**Ref**	**Ref**	**Ref**
Peri-urban	1.22 (0.74–2.01) 0.446	1.02 (0.55–1.87) 0.961	0.90 (0.48–1.71) 0.759
Rural	0.78 (0.28–2.17) 0.630	0.84 (0.20–2.03) 0.445	1.01 (0.32–3.24) 0.981
Employment status			
No	**Ref**	**Ref**	**Ref**
Yes	0.84 (0.56–1.26) 0.398	0.77 (0.48–1.24) 0.286	0.78 (0.47–1.27) 0.311
Average monthly income			
<500	**Ref**	**Ref**	**Ref**
500–1,000	1.09 (0.74–1.61) 0.671	0.97 (0.62–1.54) 0.916	0.95 (0.59–1.53) 0.843
1,001–2,000	0.92 (0.53–1.59) 0.772	0.95 (0.51–1.79) 0.886	0.99 (0.51–1.91) 0.980
2,001–5,000	0.85 (0.48–1.50) 0.569	0.88 (0.46–1.69) 0.704	1.12 (0.57–2.18) 0.749
>5,000	0.59 (0.27–1.29) 0.186	0.48 (0.20–1.23) 0.130	0.75 (0.30–1.91) 0.552
Symptoms			
1 only	**Ref**	**Ref**	**Ref**
2+	1.31 (0.92–1.87) 0.134	1.24 (0.81–1.89) 0.316	1.28 (0.84–1.97) 0.250
Initial facility visited			
Health facility	**Ref**	**Ref**	**Ref**
Other places	2.77 (1.67–4.59) **<0.001**	3.24 (1.68–6.25) **<0.001**	3.76 (2.02–7.02) **<0.001**
Number of health facilities previously visited			
None	**Ref**	**Ref**	**Ref**
1 facility	0.55 (0.31–0.98) **0.044**	0.61 (0.32–1.16) 0.128	0.46 (0.24–0.88) **0.019**
2 facilities	0.50 (0.27–0.93) **0.027**	0.53 (0.26–1.05) 0.067	0.43 (0.21–0.85) **0.016**
3+ facilities	0.60 (0.29–1.24) 0.171	0.82 (0.36–1.87) 0.638	0.49 (0.21–1.14) 0.097
Tumour stage			
Stage I&II	**Ref**	**Ref**	**Ref**
Stage III & IV	2.55 (1.82–3.58) **<0.001**	2.70 (1.84–3.96) **<0.001**	2.36 (1.57–3.54) **<0.001**

Significance was set at *p*-value < 0.05. All the bold values are less than 0.05 and therefore statistically significant.

**Table 3.** Multivariate ordinal and logistic regression showing factors associated with breast cancer among study participants with less than two months delay before visiting treatment facility with the onset of symptoms.

Variable	Delay in months		
	Model performance for binary outcomes		
	Model 4 4 months delay	Model 5 5 months delay	Model 6 6 months delay
	aOR (95%CI) *p*-value	aOR (95%CI) *p*-value	aOR (95%CI) *p*-value
Age			
≤39	**Ref**	**Ref**	**Ref**
40–49	0.62 (0.31–1.24) 0.178	0.5 (0.24–1.04) 0.063	0.49 (0.23–1.06) 0.071
50–59	0.76 (0.38–1.50) 0.429	0.77 (0.38–1.56) 0.477	0.75 (0.35–1.58) 0.446
60+	0.61 (0.28–1.29) 0.195	0.52 (0.23–1.14) 0.104	0.65 (0.28–1.50) 0.314
Religion			
Christianity	**Ref**	**Ref**	**Ref**
Islam	0.97 (0.43–2.16) 0.941	0.84 (0.36–1.98) 0.694	0.90 (0.37–2.20) 0.818
Educational level			
No formal Education	**Ref**	**Ref**	**Ref**
Primary	0.93 (0.35–2.12) 0.892	0.79 (0.28–2.20) 0.651	0.84 (0.29–2.44) 0.748
Middle /Junior High School	0.89 (0.39–2.05) 0.789	0.74 (0.32–1.74) 0.496	0.79 (0.32–1.94) 0.606
Secondary/Senior High School	1.21 (0.52–2.82) 0.652	1.15 (0.49–2.70) 0.750	1.32 (0.54–3.23) 0.548
Tertiary	0.91 (0.36–2.29) 0.846	0.75 (0.29–1.93) 0.544	0.80 (0.29–2.20) 0.669
Marital status			
Never married	**Ref**	**Ref**	**Ref**
Married	1.41 (0.69–2.86) 0.344	1.57 (0.73–3.39) 0.250	1.40 (0.63–3.11) 0.409
Divorced/Separated	2.10 (0.93–4.72) 0.072	2.14 (0.89–5.11) 0.087	1.69 (0.68–4.23) 0.259
Widowed	1.36 (0.56–3.29) 0.491	1.57 (0.61–4.06) 0.352	1.72 (0.65–4.54) 0.275
Place of residence			
Urban	**Ref**	**Ref**	**Ref**
Peri-urban	0.84 (0.40–1.75) 0.638	1.05 (0.50–2.20) 0.901	1.15 (0.54–2.47) 0.719
Rural	1.38 (0.40–3.29) 0.611	1.17 (0.30-4.53) 0.822	0.80 (0.17–3.81) 0.778
Employment status			
No	**Ref**	**Ref**	**Ref**
Yes	0.57 (0.33–0.96) **0.037**	0.65 (0.37–1.14) 0.135	0.82 (0.45–1.48) 0.511
Average monthly income (GHS)			
<500	**Ref**	**Ref**	**Ref**
500–1,000	0.91 (0.54–1.53) 0.717	0.93 (0.54–1.61) 0.792	0.89 (0.50–1.57) 0.689
1,001–2,000	0.81 (0.39–1.68) 0.565	0.77 (0.36–1.69) 0.522	0.68 (0.30–1.54) 0.356
2,001–5,000	0.94 (0.45–1.99) 0.879	0.86 (0.39–1.93) 0.720	0.66 (0.28–1.57) 0.349
>5,000	0.62 (0.20–1.89) 0.402	0.78 (0.25–2.42) 0.671	0.63 (0.19–2.14) 0.464
Symptoms			
1 only	**Ref**	**Ref**	**Ref**
2+	1.08 (0.67–1.74) 0.751	0.93 (0.56–1.55) 0.782	1.02 (0.60–1.73) 0.955
Initial facility visited			
Health facility	**Ref**	**Ref**	**Ref**
Other places	2.45 (1.33–4.52) **0.004**	2.24 (1.20–4.19) **0.012**	1.91 (0.99–3.70) 0.054
Number of health facilities previously visited			
None	**Ref**	**Ref**	**Ref**
1 facility	0.51 (0.26–1.01) 0.053	0.59 (0.29–1.19) 0.140	0.51 (0.25–1.04) 0.065
2 facilities	0.41 (0.19–0.85) **0.017**	0.44 (0.20–0.96) **0.039**	0.39 (0.177–0.86) **0.021**
3+ facilities	0.37 (0.14–0.95) **0.038**	0.31 (0.11–0.89) **0.029**	0.30 (0.10–0.90) **0.031**
Tumour stage			
Stage I & II	**Ref**	**Ref**	**Ref**
Stage III & IV	2.31 (1.45–3.69) **<0.001**	2.16 (1.32–3.54) **0.002**	1.73 (1.04–2.88) **0.033**

Significance was set at *p*-value < 0.05. All the bold values are less than 0.05 and therefore statistically significant.

**Table 4.** Reasons for patient delays.

Variable		Delay in months				*p*-value
	<1	1–2	3–6	> 6	Total (%)	
Advice from family/friends						**0.010**
No	214 (79.8)	125 (83.9)	120 (91.6)	79 (89.8)	538 (84.6)	
Yes	54 (20.2)	24 (16.1)	11 (8.4)	9 (10.2)	98 (15.4)	
Lack of knowledge of breast cancer symptoms						**<0.001**
No	213 (79.5)	61 (40.9)	43 (32.8)	21 (23.9)	338 (53.1)	
Yes	55 (20.5)	88 (59.1)	88 (67.2)	67 (76.1)	298 (46.9)	
Health system factors (including appointment system)						0.204
No	252 (94.0)	138 (92.6)	128 (97.7)	85 (96.6)	603 (94.8)	
Yes	16 (6.0)	11 (7.4)	3 (2.3)	3 (3.4)	33 (5.2)	
Financial issues						**0.003**
No	256 (95.5)	127 (85.2)	117 (89.3)	78 (88.6)	578 (90.9)	
Yes	12 (4.5)	22 (14.8)	14 (10.7)	10 (11.4)	58 (9.1)	
Competing priorities (work, family, school)						**0.014**
No	260 (97.0)	133 (89.3)	121 (92.4)	83 (94.3)	597 (93.9)	
Yes	8 (3.0)	16 (10.7)	10 (7.6)	5 (5.7)	39 (6.1)	
Nobody to come to hospital with						0.525
No	266 (99.2)	146 (98.0)	130 (99.2)	86 (97.7)	628 (98.7)	
Yes	2 (0.8)	3 (2.0)	1 (0.8)	2 (2.3)	8 (1.3)	
Fear of treatment						0.164
No	260 (97.0)	147 (98.7)	123 (93.9)	85 (96.6)	615 (96.7)	
Yes	8 (3.0)	2 (1.3)	8 (6.1)	3 (3.4)	21 (3.3)	
Sought alternate healing (Including faith based)						**0.001**
No	263 (98.1)	135 (90.6)	111 (84.7)	81 (92.0)	590 (92.8)	
Yes	5 (1.9)	14 (9.4)	20 (17.3)	7 (7.8)	46 (7.2)	
Indifference						**0.038**
No	260 (97.0)	136 (91.3)	124 (94.7)	86 (97.7)	606 (95.3)	
Yes	8 (3.0)	13 (8.7)	7 (5.3)	2 (2.3)	30 (4.7)	
Did not believe the diagnosis						0.818
No	263 (98.1)	144 (96.6)	128 (97.7)	86 (97.7)	621 (97.6)	
Yes	5 (1.9)	5 (3.4)	3 (2.3)	2 (2.3)	15 (2.4)	
